# Fusing Artificial Intelligence and Machine Learning for Anti-Cancer Drug Discovery

**DOI:** 10.3390/cancers16203522

**Published:** 2024-10-17

**Authors:** Christos Adamopoulos, Kostas A. Papavassiliou, Athanasios G. Papavassiliou

**Affiliations:** 1 Department of Biological Chemistry, Medical School, National and Kapodistrian University of Athens, 11527 Athens, Greece; 2 Department of Oncological Sciences, Icahn School of Medicine at Mount Sinai, New York, NY 10029, USA; 3 First University Department of Respiratory Medicine, ‘Sotiria’ Chest Hospital, Medical School, National and Kapodistrian University of Athens, 11527 Athens, Greece; konpapav@med.uoa.gr

The integration of artificial intelligence (AI) and machine learning (ML) in modern oncology is rapidly transforming cancer drug discovery and development. These technologies enable scientists to overcome obstacles in the traditional drug development pipeline, accelerating the discovery and optimization of new anti-cancer therapies. AI’s ability to simulate human cognitive processes, in combination with ML, offers new opportunities for identifying novel drug targets, predicting patient responses, and conquering cancer drug resistance [1,2,3,4].

AI models using deep learning have already shown success in accurately predicting drug sensitivity and resistance across various cancer types, enabling more personalized treatment approaches [1]. The role of AI in identifying novel drug targets has also gained momentum. By mapping biological networks and chartographing intricate molecular circuits, AI can pinpoint previously undiscovered interactions within cell systems, revealing new potential therapeutic targets. Despite challenges like high computational costs and data bias, ML approaches such as graph convolution networks are advancing target identification and improving drug property predictions by analyzing biomolecular structures and clinical data [2]. Furthermore, AI has demonstrated success using a deep learning model, POLYpharmacology Generative Optimization Network (POLYGON), in designing compounds that can inhibit more than one target simultaneously [3]. In addition, models like Drug Ranking Using ML (DRUML) have been developed to rank drugs, employing large-scale “omics” data and predicting their efficacy performance across diverse cancer types [4]. Deep learning models such as AlphaFold (see below) have further revolutionized the field by accurately predicting protein structures and interactions, which are essential for designing drugs that precisely target cancer-related proteins [5,6].

The potential of AI extends to drug screening and the repurposing of drugs. Advanced AI tools like PockDrug predict “druggable” pockets on proteins, whilst AlphaFold and other structural biology models further refine these predictions, helping identify new drugs and repurposing existing ones for cancer treatment [2]. Notwithstanding some limitations, such as the complexity of protein dynamics and the need for more efficient feature selection algorithms, AI is steadily ameliorating these predictions and driving drug discovery forward [2,5]. Moreover, AI has proven valuable in understanding the mechanisms of drug resistance in cancer. A notable application is in breast cancer research, where deep learning models have elucidated the mechanisms for resistance to cyclin-dependent kinase 4 and 6 (CDK4/6) inhibitors, providing potential overcoming treatment strategies [7]. AI’s transformative impact is evident in its capacity to accelerate discoveries in unexplored areas. Crowdsourced efforts have expanded the understanding of kinase inhibitor interactions, revealing new targets within the human kinome [8]. Similarly, AI models integrated with multi-scale interactome networks have provided insights into how drugs affect biological systems, aiding in the prediction of treatment outcomes more accurately [9].

The development of AI-powered platforms like canSAR, the world’s largest public cancer drug discovery knowledgebase, has further propelled this field. CanSAR integrates data from various sources to fuel AI-driven models, enhancing the efficiency and accuracy of drug discovery efforts [10]. AlphaFold has also made significant contributions to the structural prediction of proteins, offering critical insights into how cancer-related proteins (e.g., tumor-cell signal transduction components and effectors) interact with drugs and open up new pathways for treatment [11]. While these advancements are promising, several challenges remain. Data quality and biases can affect the accuracy of AI models, and large datasets are needed to train these algorithms effectively [12]. However, initiatives aimed at improving data collection and sharing will continue to foster the performance of AI-driven models [13].

The above findings are emphatically certified by the awarding of the 2023 Albert Lasker Basic Medical Research Award and the 2024 Nobel Prize in Chemistry to the development of an AI model, namely AlphaFold, to predict protein structures and design our own proteins, a colossal achievement that is likely to dramatically expedite cancer drug development because it will abate the length of the iteration cycle of therapeutic development and decode the mechanisms of action. Such predictive models will also allow much finer-grained and more causal interpretation of a patient’s genomic data, revealing how individual genetic variations impact on cancer cell behavior and possibly suggesting a route toward more personalized medicine [14].
